# Supplementary material for: Identification of photoperiod-induced specific miRNAs in the adrenal glands of Sunite sheep (Ovis aries)
Source: Front Vet Sci. 2022 Jul 22;9:888207. doi: 10.3389/fvets.2022.888207 (PMC9354845; doi:10.3389/fvets.2022.888207)
Supplement: Supplementary file 1 [file Data_Sheet_1.ZIP › Supplementary file/miRDeep2 file.zip!/miRDeep2 file/Novel_Structure/SP42A2/Novel_39.pdf]

Provisional ID	:	NC_019460.2_9346
Score total	:	323.9
Score for star read(s)	:	3.9
Score for read counts	:	316.2
Score for mfe	:	2.1
Score for randfold	:	1.6
Score for cons. seed	:	
Total read count	:	632
Mature read count	:	620
Loop read count	:	0
Star read count	:	12

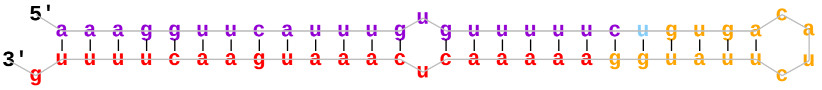

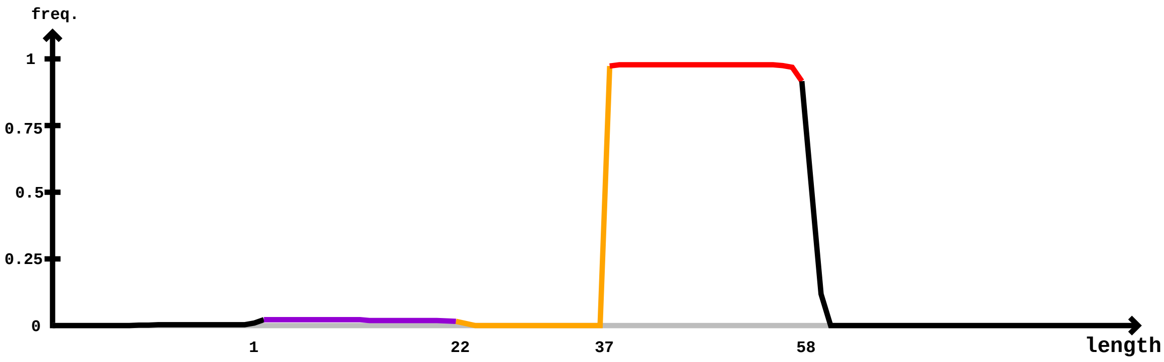

Star Mature

```
5'- aaauaauacuggguuggccaaaagguucauuuguguuuuucugugacaucuuaugguaaaaacucaaaugaacuuuugggccaacccaaugaaaagcuccaaucccuucacu  -3'   obs
    aaauaauacuggguuggccaaaagguucauuuguguuuuucugugacaucuuaugguaaaaacucaaaugaacuuuugggccaacccaaugaaaagcuccaaucccuucacu         exp
    ..........(((((((((.(((((((((((((.((((((((((((....)))))))))))).))))))))))))).))))))))).........................   reads   mm   sample
    .......acuggguuggccaaaaCguucauu................................................................................   1       1    seq
    .........uggguuggccaaaaCguucauu................................................................................   1       1    seq
    ...................aaaagguucauuuguguuuu........................................................................   1       0    seq
    ...................aaaagguucauuugGguuuu........................................................................   1       1    seq
    ...................aaaagguucauuuguguuuuuc......................................................................   2       0    seq
    ....................aaagguucauuuguguuuuuc......................................................................   7       0    seq
    ....................aaagguucauuugAguuuuucu.....................................................................   1       1    seq
    ........................................................aaaaacucaaaugaacuu.....................................   1       0    seq
    ........................................................aaaaacCcaaaugaacuu.....................................   1       1    seq
    ........................................................aaaaacucaGaugaacuuu....................................   1       1    seq
    ........................................................aaaaacucaaaugaacuuu....................................   3       0    seq
    ........................................................aaaaacCcaaaugaacuuuu...................................   1       1    seq
    ........................................................aaaaacucaaaugaacuuuu...................................   30      0    seq
    ........................................................aGaaacucaaaugaacuuuu...................................   1       1    seq
    ........................................................aaaaacucaaaugaacuuuG...................................   1       1    seq
    ........................................................aGaaacucaaaugaacuuuug..................................   1       1    seq
    ........................................................aaaaacucaaaugaacuuuug..................................   456     0    seq
    ........................................................aaaaGcucaaaugaacuuuug..................................   4       1    seq
    ........................................................aaaaacucaaaugaacuuuuA..................................   36      1    seq
    ........................................................aaaaacucaaaugaacCuuug..................................   1       1    seq
    ........................................................aaaaacucaaaGgaacuuuug..................................   1       1    seq
    ........................................................aaaaacucaaaCgaacuuuug..................................   5       1    seq
    ........................................................aaaaacucaGaugaacuuuug..................................   2       1    seq
    ........................................................aaaaacucaaaugaacuuuuggC................................   11      1    seq
    ........................................................aaaaacucaaaugaacuuuuggU................................   61      1    seq
    .........................................................aaaacucaaaugaacuuuuggU................................   3       1    seq
```
